# Airway epithelial cell-specific deletion of HMGB1 exaggerates inflammatory responses in mice with muco-obstructive airway disease

**DOI:** 10.3389/fimmu.2022.944772

**Published:** 2023-01-19

**Authors:** Yun Mao, Sonika Patial, Yogesh Saini

**Affiliations:** Department of Comparative Biomedical Sciences, School of Veterinary Medicine, Louisiana State University, Baton Rouge, LA, United States

**Keywords:** HMGB1, cystic fibrosis, airway epithelium, mucus obstruction, *Scnn1b*-Tg+

## Abstract

High mobility group box 1 (HMGB1), a ubiquitous chromatin-binding protein required for gene transcription regulation, is released into the extracellular microenvironment by various structural and immune cells, where it is known to act as an alarmin. Here, we investigated the role of airway epithelium-specific HMGB1 in the pathogenesis of muco-obstructive lung disease in *Scnn1b*-transgenic (Tg+) mouse, a model of human cystic fibrosis (CF)-like lung disease. We hypothesized that airway epithelium-derived HMGB1 modulates muco-inflammatory lung responses in the Tg+ mice. The airway epithelium-specific HMGB1-deficient mice were generated and the effects of HMGB1 deletion on immune cell recruitment, airway epithelial cell composition, mucous cell metaplasia, and bacterial clearance were determined. The airway epithelium-specific deletion of HMGB1 in wild-type (WT) mice did not result in any morphological alterations in the airway epithelium. The deficiency of HMGB1 in airway epithelial cells in the Tg+ mice, however, resulted in significantly increased infiltration of macrophages, neutrophils, and eosinophils which was associated with significantly higher levels of inflammatory mediators, including G-CSF, KC, MIP-2, MCP-1, MIP-1α, MIP-1β, IP-10, and TNF-α in the airspaces. Furthermore, as compared to the HMGB1-sufficient Tg+ mice, the airway epithelial cell-specific HMGB1-deficient Tg+ mice exhibited poor resolution of spontaneous bacterial infection. The HMGB1 deficiency in the airway epithelial cells of Tg+ mice did not alter airway epithelial cell-specific responses including epithelial cell proliferation, mucous cell metaplasia, and mucus obstruction. Collectively, our findings provide novel insights into the role of airway epithelial cell-derived HMGB1 in the pathogenesis of CF-like lung disease in Tg+ mice.

## Introduction

Muco-obstructive lung diseases, including cystic fibrosis (CF), are characterized by muco-obstructive airways, increased levels of pro-inflammatory mediators, and infiltration of immune cells (1, 2). These alterations contribute to an increase in the percent solid contents of the airway surface liquid (ASL) layer leading to compromised functioning of the mucociliary clearance (MCC) apparatus which, in turn, contributes to the increased susceptibility of muco-obstructive lung disease patients to microbial infections (3). In response to the prevailing stress, the airway epithelial cells are known to release damage-associated molecular patterns (DAMPs) such as high mobility group box protein 1 (HMGB1) (4, 5), interleukin 33 (IL-33) (6), interleukin 25 (IL-25) (7), thymic stromal lymphopoietin (TSLP) (7), interleukin 1α (IL-1α) (8), heat shock proteins (HSPs) (9, 10), and calprotectin (S100A8/9) (11). However, the roles of these molecules in the pathogenesis of muco-obstructive lung diseases are not fully understood.

HMGB1, a ubiquitous chromatin-binding protein, is required for nucleosome stability and gene transcription regulation (12). In addition, upon its release into the stressed extracellular microenvironment by various structural and immune cells, HMGB1 is known to play a role as an alarmin (13, 14). HMGB1 also acts as a pro-inflammatory mediator in various inflammatory diseases (15–17). HMGB1 levels are upregulated in the sputum and bronchoalveolar lavage fluid (BALF) of CF patients and cystic fibrosis mice models (17–21). In addition, HMGB1 expression is increased in the pulmonary epithelial cells in murine models of chronic asthma (22, 23) and acute lung injury (24), suggesting that these cells are the likely origins of the secreted HMGB1 in the BALF. Accordingly, we investigated the role of airway epithelial cell-derived HMGB1 in the pathogenesis of muco-obstructive lung disease in *Scnn1b*-transgenic (*Scnn1b*-Tg+) mouse, a model of human CF-like lung disease.

The *Scnn1b*-Tg+ (Tg+) mouse overexpresses sodium channel non-voltage gated 1, β subunit (*Scnn1b*) transgene in the airway epithelial club cells, resulting in the overexpression of its translational product, i.e., epithelial Na (+) channel subunit β (25). The overexpression of *Scnn1b* transgene increases the transport of sodium ions as well as water into the airway epithelial cells, which results in the ASL dehydration. As a result, the Tg+ mice exhibit various features of muco-obstructive airway diseases including mucus obstruction, mucous cell metaplasia, lung inflammation, and bacterial infection (2, 25–30). The Tg+ neonates are prone to early spontaneous bacterial infections that are cleared almost completely by the adulthood (26, 31). HMGB1 levels are elevated in the BALF of Tg+ mice (18). However, the sources of the increased amount of HMGB1 in the BALF and its role in the muco-inflammatory responses in the Tg+ mice remain unclear.

We hypothesized that airway epithelial cell-derived HMGB1 modulates muco-inflammatory responses in the Tg+ mice. To test our hypothesis, airway epithelial cell-specific HMGB1-deficient Tg+ mice were generated and examined for alterations in muco-obstructive responses in Tg+ lungs. Because the Tg+ neonates (5-10 days old) exhibit robust type 1 inflammation with spontaneous bacterial infection and, on the other hand, the Tg+ adults exhibit robust muco-obstructive phenotype with almost no bacterial infections, we employed juvenile mice (3-week-old) that exhibit most of the hallmarks of Tg+ lung disease, i.e., bacterial infections, muco-obstructive, mucous cell metaplasia, and mixed granulocytic inflammation of Tg+ mice (26, 28–30). Specifically, the effects of airway epithelial cell-specific HMGB1 deletion on key endpoints including immune cell recruitment, airway epithelial cell composition, mucus obstruction, and bacterial clearance were examined. The results from this study provide novel insights into the role of airway epithelial cell-derived HMGB1 in the pathogenesis of CF-like lung disease in Tg+ mice.

## Materials and methods

### Generation of mice strains and animal husbandry

Three mice strains, i.e., club cell-specific Cre recombinase (CCSP-Cre^+^) strain (a kind gift by Dr. Francesco J. DeMayo, NIEHS) (32), floxed *Hmgb1* (*Hmgb1*^fl/fl^) strain (RBRC06240; Riken BioResource Research Center, Ibaraki, Japan) (33), and *Scnn1b*-Tg+ (Tg+) mice [Stock Number, 006438; Strain name, B6N.Cg-Tg(Scgb1a1-Scnn1b)6608Bouc/J, The Jackson Laboratory, Bar Harbor, ME] were interbred to generate parental strains. Airway epithelial cell-specific HMGB1-deficient Tg+ (CCSP-Cre^+^/*Hmgb1*^fl/fl^/Tg+) mice, HMGB1-sufficient Tg+ (CCSP-Cre^-^/*Hmgb1*^fl/fl^*/*Tg+) mice, and their non-transgenic counterparts (CCSP-Cre^-^/*Hmgb1*^fl/fl^*/*WT mice and CCSP-Cre^+^/*Hmgb1*^fl/fl^*/*WT mice) were generated by crossing CCSP-Cre^+^/*Hmgb1*^fl/fl^/WT and CCSP-Cre^-^/*Hmgb1*^fl/fl^/Tg+ (or CCSP-Cre^+^/*Hmgb1*^fl/fl^/Tg+ and CCSP-Cre^-^/*Hmgb1*^fl/fl^/WT) parental strains. Genotype status of the three genes in all the experimental mice were determined by polymerase chain reaction (PCR) (34, 35). Primer sequences used for genotyping are included in **Supplemental Table 1**. Animals were housed in hot-washed ventilated cages at the Division of Laboratory Animal Medicine (DLAM) of Louisiana State University (LSU) on a 12-hour day/night cycle and were provided food and water *ad libitum*. All the animal experiments were approved by the LSU Institutional Animal Care and Use Committee (IACUC).

### BALF and tissue collection

Three-week-old (postnatal day 20-23) weanlings were anesthetized *via* intraperitoneal administration of 2,2,2-tribromoethanol (Sigma-Aldrich, St. Louis, MO). Bronchoalveolar lavage fluid (BALF) was aseptically harvested from the right lungs by lavaging with calcium- and magnesium-free Dulbecco’s Phosphate Buffered Saline (DPBS) (Corning, Manassas, VA). Cell-free BALF samples were collected after centrifugation at 500 × *g* for 5 min at 4°C and stored at -80°C for estimation of total protein concentration, total dsDNA contents, HMGB1 contents, and cytokine levels. The BALF cell pellets were re-suspended in 500 µl of DPBS and used for the determination of counts and proportions of immune cells, as previously reported (36). The unlavaged left lungs were stored in 10% neutral buffered formalin and processed for histopathological evaluation. The lavaged right lungs were snap-frozen in liquid nitrogen and subsequently stored at -80°C for gene expression analyses.

### Cytokine detection in BALF

Levels of mouse cytokines and chemokines were determined in cell-free BALF supernatant by a multiplex immunoassay (MCYTOMAG-70K, EMD Millipore, Billerica, MA), according to the manufacturer instructions. The list of the cytokines and chemokines is included in **Supplemental Table 2**.

### Measurement of gene expression

Total RNA isolation, cDNA generation, and reverse transcription-polymerase chain reaction (RT-PCR) were performed as previously described (36). The primer sequences used in RT-PCR are included in **Supplemental Table 1**.

### Determination of bacterial burden

For each animal, 100 µl of diluted aseptically harvested BALF was plated onto Columbia blood agar plates (Hardy Diagnostics, Santa Maria, CA). Then the plates were incubated at 37°C in an anaerobic candle jar for 24-48 h. The colony-forming units (CFUs) were counted and morphological characteristics of colonies including size, shape, color, and margins were recorded, as previously described (34, 35).

### Histopathology examination

The unlavaged left lung lobes were fixed in formalin (10% neutral buffered formalin), paraffin-embedded, and sectioned. Alcian blue/periodic acid Schiff (AB/PAS) staining was performed to assess the presence of intracellular and extracellular mucopolysaccharides. The mucoobstruction assessment was graded by a blinded board-certified anatomic pathologist using the histological semiquantitative grading strategy, as described previously (34, 36).

### Immunohistochemistry

Five-micrometer thick, formalin-fixed, paraffin-embedded lung sections were immunohistochemically analyzed for the expression of HMGB1, forkhead box J1 (FOXJ1), club-cell secretory protein (CCSP), mucin 5AC (MUC5AC), mucin 5B (MUC5B), keratin 5 (KRT5), and KI-67. The sections were stained with the corresponding primary antibodies: rabbit polyclonal HMGB1 antibody (ab18256; Abcam, Cambridge, MA), rabbit monoclonal FOXJ1 antibody (ab235445; Abcam, Cambridge, MA), rabbit monoclonal uteroglobin antibody (ab213203; Abcam, Cambridge, MA), rabbit polyclonal MUC5AC antibody (UNC 294, a kind gift by Dr. Camille Ehre, University of North Carolina, Chapel Hill, NC), rabbit polyclonal MUC5B antibody (UNC223, a kind gift by Dr. Camille Ehre, University of North Carolina, Chapel Hill, NC), rabbit monoclonal Cytokeratin 5 antibody (ab52635; Abcam, Cambridge, MA), and rabbit monoclonal KI-67 antibody (ab16667; Abcam, Cambridge, MA), as published previously (34, 35).

### Western blotting

Equal volumes of BALF were subjected to electrophoresis on NuPAGE 4-12% Bis-Tris gradient gel (Life Technologies, Carlsbad, CA), and transferred to PVDF membrane by using iBlot™ gel transfer device (Life Technologies, Carlsbad, CA). Then, the membrane was incubated with rabbit polyclonal HMGB1 primary antibody (ab18256; Abcam, Cambridge, MA). Alexa Fluor 680 goat anti-rabbit IgG (926-68071; LI-COR Biosciences, Lincoln, NE) was used as the secondary antibody. Protein bands were visualized using Odyssey CLx, Imager (LI-COR Biosciences, Lincoln, NE).

### Statistical analyses

One way analysis of variance (ANOVA) test followed by Tukey’s *post hoc* test was used for the statistical analysis among groups. Data were presented as Mean ± Standard Error of the Mean (SEM) and Grubbs’ test was used to identify outliers. A *p* value of less than 0.05 was considered statistically significant. All statistical analyses were performed using the program GraphPad Prism 9.0 (GraphPad Software, Inc., La Jolla, CA).

## Results

### Airway epithelial cell-specific HMGB1-deficiency results in a significant reduction in the HMGB1 secretion into the healthy airspaces

**Figure 1:**
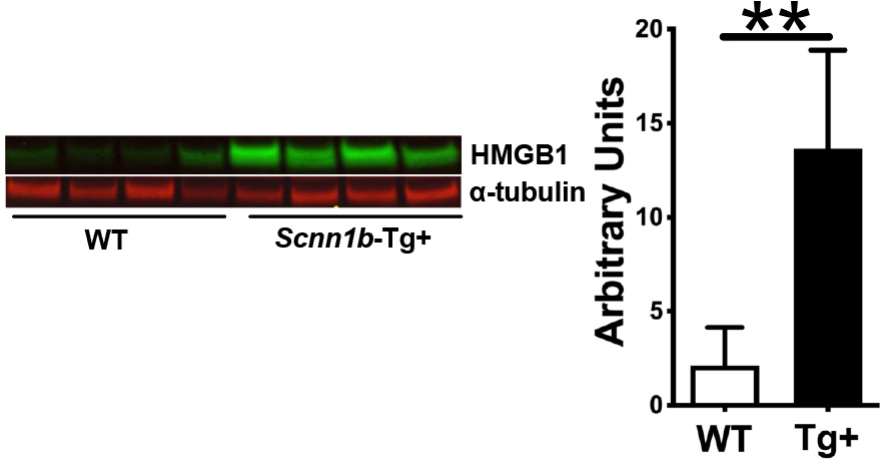
HMGB1 expression is up-regulated in the BALF of Scnn1b-Tg+ (Tg+) lungs. The representative immunoblots (left) and corresponding quantification of the immunoblots (right). Equal volumes of cell-free BALF from WT and Tg+ adult mice (n=4/group) were used. α-tubulin was used as a loading control. Error bars represent Mean ± SEM. One-way ANOVA followed by Tukey’s *post hoc* test was used for the statistical analysis. **p < 0.01.

**Figure 2:**
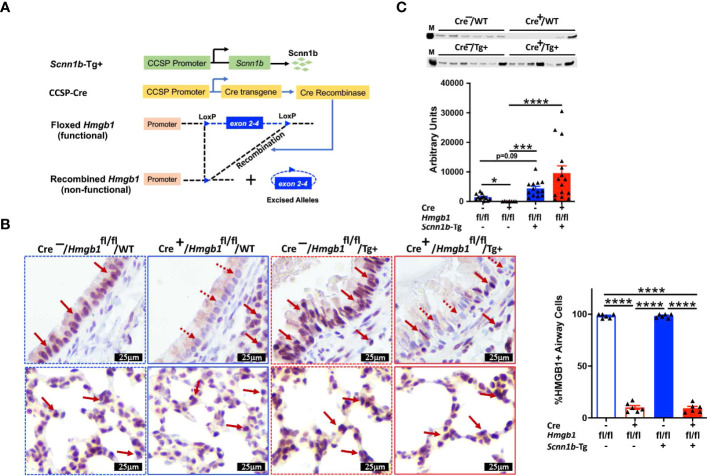
Airway epithelial cell-specific deletion of HMGB1. **(A)** Schematic diagram of transgenes used in the generation of airway epithelial cell-specific HMGB1-deficient mice. Airway epithelial cell-specific HMGB1-deficient *Scnn1b*-Tg+ (CCSP-Cre^+^/*Hmgb1*^fl/fl^/Tg+) mice were generated by crossing club cell-specific Cre recombinase (CCSP-Cre^+^), floxed *Hmgb1* (*Hmgb1*^fl/fl^), and *Scnn1b*-Tg+ mice. **(B)** Immunohistochemistry for HMGB1 in lung sections from airway epithelial cell-specific HMGB1-sufficient and airway epithelial cell-specific HMGB1-deficient WT and Tg+ mice. Red solid arrow indicates the HMGB1-stained cells. Red dotted arrow indicates the cells negatively stained for HMGB1. Bar graph shows percent HMGB1-stained cells in the airways. Sample size n=6/group; Error bars represent Mean ± SEM. One-way ANOVA followed by Tukey’s *post hoc* test was used for the statistical analysis. ****p < 0.0001. **(C)** Western blot showing the depletion of HMGB1 in Cre^+^/*Hmgb1*^fl/fl^/WT (Cre^+^/WT) juveniles (red open bar), while comparable HMGB1 between Cre^-^*Hmgb1*^fl/fl^/Tg+ (Cre^-^/Tg+) (blue solid bar) and Cre^+^/*Hmgb1*^fl/fl^/Tg+ (Cre^+^/WT) (red solid bar) juveniles. Equal volumes of cell-free BALF from all the groups were used as loading samples. M, band from size ladder. Sample size n=7-15/group. Error bars represent Mean ± SEM. One-way ANOVA followed by Tukey’s *post hoc* test was used for the statistical analysis. *p < 0.05, ***p < 0.001, ****p < 0.0001.

First, we analyzed the BALF HMGB1 protein levels in WT and Tg+ mice (**Figure 1**). As compared with WT mice, HMGB1 contents were significantly increased in cell-free BALF of the Tg+ mice (**Figure 1**). Next, we hypothesized that the airway epithelial cells release HMGB1 in response to the ASL dehydration and that the deficiency of HMGB1 in airway epithelial cells will modulate the muco-inflammatory pathology in Tg+ mice. Since whole-body HMGB1 deletion is embryonically lethal (37, 38), we employed the Cre-LoxP-based airway epithelial cell-specific deletion approach. We generated airway epithelial cell-specific HMGB1-deficient Tg+ mice by crossing Tg+, club cell-specific Cre recombinase (CCSP-Cre^+^), and floxed *Hmgb1* (*Hmgb1*^fl/fl^) strains (**Figure 2A**). First, to ascertain the deletion of HMGB1 in CCSP-Cre^+^/*Hmgb1*^fl/fl^ mice, we performed HMGB1 immunohistochemical staining on lung sections from these mice. While the airway epithelial cells in CCSP-Cre^-^/*Hmgb1*^fl/fl^/WT (Cre^-^/WT) and CCSP-Cre^-^/*Hmgb1*^fl/fl^/*Scnn1b*-Tg+ (Cre^-^/Tg+) mice had consistent nuclear staining for HMGB1 (**Figure 2B**, top panels), the airway epithelial cells in CCSP-Cre^+^/*Hmgb1*^fl/fl^/WT (Cre^+^/WT) and CCSP-Cre^+^/*Hmgb1*^fl/fl^/*Scnn1b*-Tg+ (Cre^+^/Tg+) mice were almost completely devoid of HMGB1 (**Figure 2B**, top panels). The staining intensity of alveolar epithelial cells was comparable between Cre^-^/WT and Cre^+^/WT mice (**Figure 2B**, bottom panels). As compared to WT mice, the staining of alveolar epithelial cells was more intense in Tg+ mice (**Figure 2B**, bottom panels). However, the HMGB1 staining of alveolar epithelial cells was comparable between Cre^-^/Tg+ and Cre^+^/Tg+ mice (**Figure 2B**, bottom panels). Next, BALF aliquots from the four groups of experimental mice were analyzed to determine the levels of HMGB1 released into the airspaces (**Figure 2C**). As expected, the HMGB1 contents were decreased in Cre^+^/WT mice when compared with Cre^-^/WT mice (**Figure 2C**). As compared with the Cre^-^/WT mice, the BALF HMGB1 levels were elevated in the Cre^-^/Tg+ mice. However, the BALF HMGB1 levels were comparable between Cre^-^/Tg+ and Cre^+^/Tg+ mice (**Figure 2C**).

### HMGB1 deletion in airway epithelial cells modulates immune cell recruitment in airspaces of Tg+ mice

Because germline HMGB1 knockout mice results in late embryonic lethality (38), we calculated mendelian ratio of progeny with expected genotype in recruited litters. The observed mendelian ratio for the Cre^-^/WT, Cre^+^/WT, Cre^-^/Tg+, and Cre^+^/Tg progeny was of 0.93:1.12:0.97:0.97, respectively. As determined from the χ^2^ value of 0.418, the observed mendelian ratio was not significantly deviated from the expected mendelian ratio of 1:1:1:1. These data suggest the airway epithelial cell-specific deletion of HMGB1 does not cause embryonic mortality. Further, the Tg+ mice exhibit neonatal mortality within the first 20 days of postnatal life (27). To determine the effect of airway epithelial cell-specific HMGB1 deficiency on the postnatal survival in WT and Tg+ mice, we observed neonatal mice until 21 days of age. The airway epithelial cell-specific HMGB1 deficiency did not cause mortality in neonates without Tg+ expression, i.e., only 1 out of 24 Cre^+^/WT neonates was found dead at the age of 4 days. As expected, the Cre^-^/Tg+ mice exhibited mortality in ~14% (3 out of 21 neonates) pups. Similarly, ~19% (4 out of 21 neonates) Cre^+^/Tg+ neonates exhibited mortality. These data suggest that the airway epithelial cell-specific deletion of HMGB1 does not cause significant increase in the mortality that is generally observed in Tg+ neonates.

**Figure 3:**
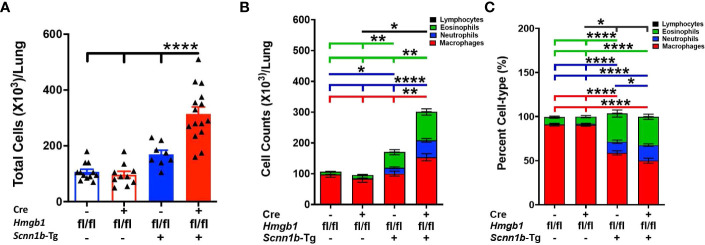
Airway epithelial cell-specific deletion of HMGB1 modulates immune cell recruitment in airspaces of Tg+ mice. Total cell counts **(A)** are shown for Cre^-^/WT [blue open bar], Cre^+^/WT [red open bar], Cre^-^/Tg+ [solid blue bar], and Cre^+^/Tg+ [solid red bar] mice). Differential cell counts **(B)** and the relative percentages **(C)** are presented as stacked bar graph (macrophages [red bar], neutrophils [blue bar], eosinophils [green bar], and lymphocytes [black bar]). The significant differences are shown by horizontal lines of cell-specific colors (macrophages [red line], neutrophils [blue line], eosinophils [green line], and lymphocytes [black line]). For enhanced clarity in the comparisons, these stacked graphs are replotted for individual cell types (**Supplemental Figure 1**). Error bars represent Mean ± SEM. One-way ANOVA followed by Tukey’s *post hoc* test was used for the statistical analysis. *p < 0.05, **p < 0.01, ****p < 0.0001.

Next, to determine the effect of airway epithelial cell-specific HMGB1 deletion on immune cell recruitment into the airspaces of Tg+ mice, we analyzed immune cells in the BALF from all the experimental groups. The total BALF cell counts trended higher in Cre^-^/Tg+ versus Cre^+^/WT and Cre^-^/WT mice (**Figure 3A**). The proportion and cell count for the four types of immune cells, i.e., macrophages, neutrophils, eosinophils, and lymphocytes were comparable between Cre^-^/WT and Cre^+^/WT mice (**Figures 3B, C** and **Supplemental Figure 1**). As compared with the Cre^+^/WT, Cre^-^/WT, and Cre^-^/Tg+ mice, the total cell counts were significantly increased in Cre^+^/Tg+ mice (**Figure 3A**). While the numbers of macrophages and lymphocytes were comparable between the Cre^-^/WT, Cre^+^/WT, and Cre^-^/Tg+ mice, the Cre^+^/Tg+ mice had significant increase in macrophages, neutrophil and eosinophil counts (**Figures 3B, C** and **Supplemental Figure 1**). This increase in total cell counts was attributed to the increased numbers of macrophages, eosinophils, and neutrophils (**Figures 3B, C** and **Supplemental Figure 1**).

### HMGB1 deletion in airway epithelial cells alters the levels of inflammatory mediators in the airspaces of Tg+ mice

**Figure 4:**
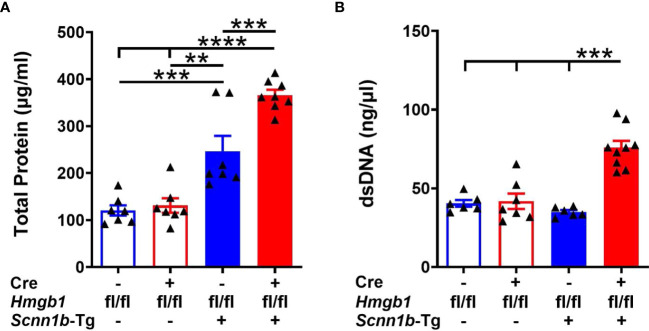
Airway epithelial cell-specific HMGB1-deficient mice exhibit elevated BALF protein and dsDNA contents in Tg+ mice. The total protein contents (µg/ml) **(A)** and dsDNA contents (ng/µl) **(B)** in cell-free BALF from WT mice (with Cre^-^ or Cre^+^ status) and Tg+ mice (with Cre^-^ or Cre^+^ status). Error bars represent Mean ± SEM. One-way ANOVA followed by Tukey’s *post hoc* test was used for the statistical analysis. Sample size n=6-9/group. **p < 0.01, ***p < 0.001, ****p < 0.0001.

Increased levels of total protein in BALF indicate injury to the endothelial-epithelial barrier in acute injury models and reflect increased inflammatory proteins in the airspaces (34, 39–41). To explore the effect of HMGB1 deletion in airway epithelial cells on BALF proteins, we examined the total proteins in BALF from WT and Tg+ mice with or without deletion of HMGB1 in the epithelial cells. The BALF total protein contents were comparable between Cre^-^/WT and Cre^+^/WT (**Figure 4A**). The total protein contents were elevated in the BALF of Cre^-^/Tg+ mice (246.8 ± 32.67 µg/ml) compared with Cre^+^/WT (131.1 ± 15.45 µg/ml) and Cre^-^/WT mice (120.6 ± 10.97 µg/ml) (**Figure 4A**). However, deletion of HMGB1 in epithelial cells significantly increased the BALF total protein in Cre^+^/Tg+ mice (366.2 ± 11.17 µg/ml; ~1.5-fold compared with Cre^-^/Tg+ mice, ~3-fold compared with Cre^-^/WT mice, and ~2.8-fold compared with Cre^+^/WT mice) (**Figure 4A**).

To determine whether the increase in the BALF protein levels in Cre^+^/Tg+ is caused due to epithelial barrier dysfunction, we assessed the mRNA levels of key apical junction complex proteins that are critical for epithelial barrier function. The mRNA levels of genes encoding tight junction (TJ) proteins, Occludins (*Ocln*), ZO1 (*Tjp1*) and adherens junction (AJ) proteins, i.e., E-Cadherin (*Cdh1*), Beta-catenin (*Ctnnb1*) were comparable between Cre^-^/Tg+ and Cre^+^/Tg+ groups (**Supplemental Figure 2**).

Increased levels of dsDNA in BALF also indicate airway epithelial cell damage and inflammation (34, 39, 40). The increased contents of the dsDNA may reflect nucleic acids associated with neutrophil extracellular trap (NETs) and nucleic acids associated with extracellular vesicles (42). To further investigate whether HMGB1 deletion in airway epithelial cells affects dsDNA release in BALF, we measured its levels in BALF from WT and Tg+ mice with or without deletion of HMGB1 in airway epithelial cells. Although no clear statistically significant differences were detected among Cre^-^/WT, Cre^+^/WT, and Cre^-^/Tg+ mice (**Figure 4B**), the deletion of HMGB1 in epithelial cells significantly increased the BALF dsDNA contents in Cre^+^/Tg+ mice compared with the three other experimental groups (**Figure 4B**).

**Figure 5:**
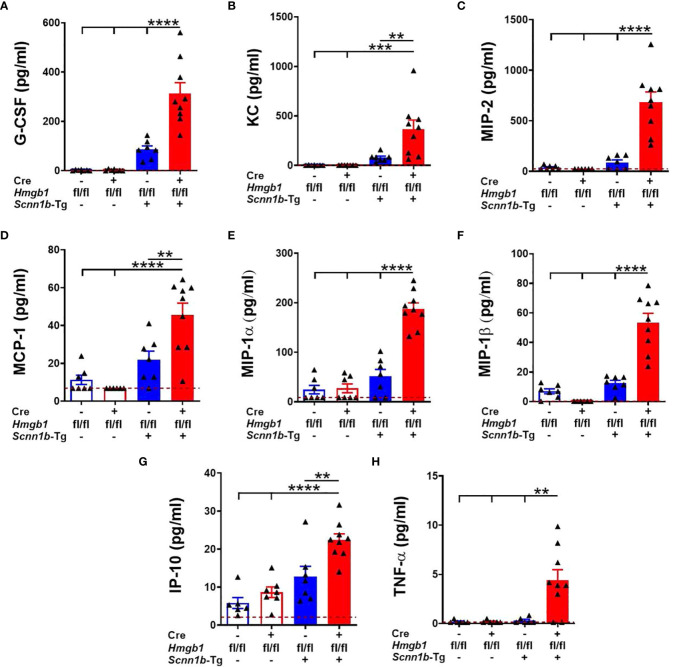
Airway epithelial cell-specific deletion of HMGB1 alters the levels of inflammatory mediators in the airspaces of Tg+ mice. Cell-free BALF cytokine levels (picograms per milliliter) of G-CSF **(A)**, KC **(B)**, MIP-2 **(C)**, MCP-1 **(D)**, MIP-1α **(E)**, MIP-1β **(F)**, IP-10 **(G)**, and TNF-α **(H)** in WT mice (with Cre^-^ or Cre^+^ status) and Tg+ mice (with Cre^-^ or Cre^+^ status) are shown. Red dotted horizontal lines indicate the lower limit of detection (LOD) obtained in the assay. The values that were below the LOD were assigned value 0.01 unit less than the LOD. Sample size n=7-9/group. Error bars represent Mean ± SEM. One-way ANOVA followed by Tukey’s *post hoc* test was used for the statistical analysis. **p < 0.01, ***p < 0.001, ****p < 0.0001.

Next, to determine the effect of airway epithelial cell-specific HMGB1 deletion on chemokines and inflammatory mediators released into the airspaces, BALF was analyzed for the levels of cytokines and chemokines (**Supplemental Table 2**). Levels of neutrophil-specific chemoattractants, i.e., G-CSF, KC/CXCL1, MIP-2/CXCL2, and MCP-1/CCL2 were comparable between the Cre^-^/WT and Cre^+^/WT mice (**Figure 5A–D** and **Supplemental Table 2**). While these four chemokines were increased in the presence of Tg+, the increases in their levels were significant in Cre^+^/Tg+ mice versus among Cre^-^/WT, Cre^+^/WT, and Cre^-^/Tg+ mice (**Figures 5A–D** and **Supplemental Table 2**). Similarly, both MIP-1α/CCL3, a chemokine for macrophages, lymphocytes, eosinophils, and neutrophils, and MIP-1β/CCL4, a chemokine for monocytes, eosinophils, and lymphocytes, were significantly increased in Cre^+^/Tg+ mice versus other three groups (**Figures 5E, F** and **Supplemental Table 2**). In addition, IP-10 (CXCL10), a chemokine for leukocytes was comparable in the BALF from Cre^-^/WT and Cre^+^/WT mice. While the Tg+ status increased the IP-10 levels in the BALF of Cre^-^/Tg+, the increase was significant only in the Cre^+^/Tg+ mice (**Figure 5G** and **Supplemental Table 2**). As compared with Cre^-^/WT, Cre^+^/WT, and Cre^-^/Tg+ mice, the TNF-α, a pro-inflammatory cytokine, was significantly elevated in the BALF of Cre^+^/Tg+ mice (**Figure 5H** and **Supplemental Table 2**). These data suggest that the airway epithelial cell-specific deficiency of HMGB1 promotes pro-inflammatory microenvironment in Tg+ airways.

### HMGB1 deletion in airway epithelial cells compromises bacterial clearance in airspaces of Tg+ mice

**Figure 6:**
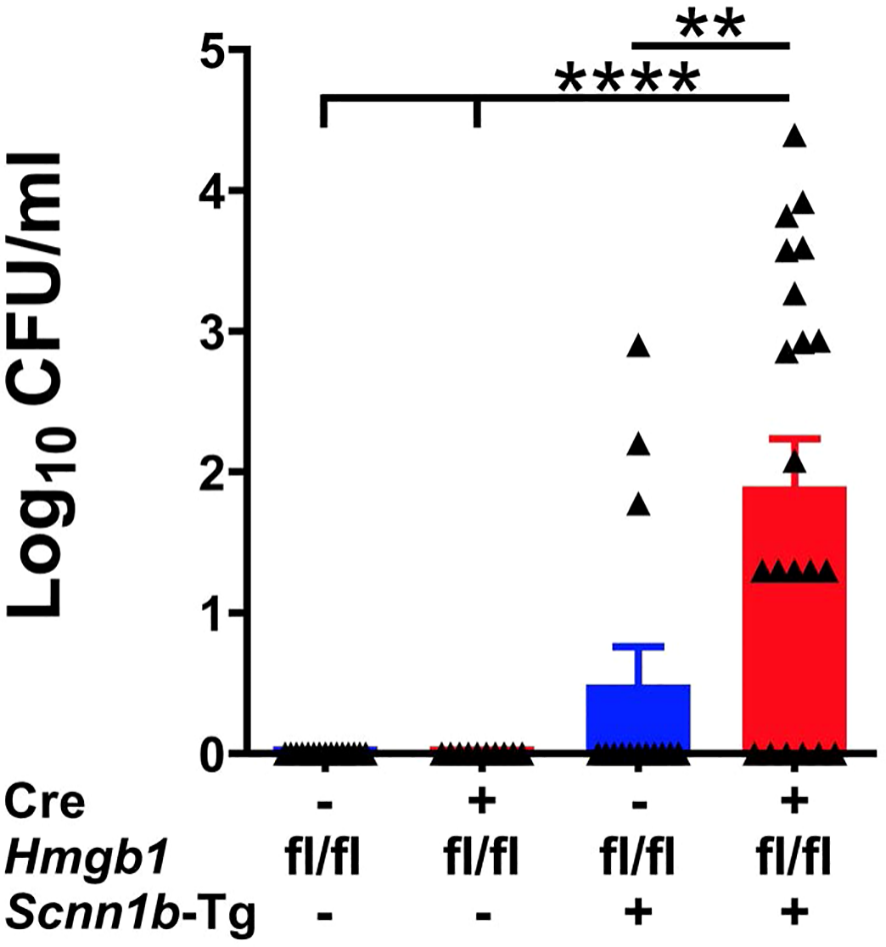
Airway epithelial cell-specific deletion of HMGB1 compromises bacterial clearance of Tg+ mice. CFUs were counted in BALF from mice with different genotypes (Cre^-^/WT [blue open bar], Cre^+^/WT [red open bar], Cre^-^/Tg+ [solid blue bar], and Cre^+^/Tg+ [solid red bar] mice). The CFU values were log_10_-transformed. Error bars represent Mean ± SEM. One-way ANOVA followed by Tukey’s *post hoc* test was used for the statistical analysis. **p < 0.01, ****p < 0.0001.

The mucostasis in the airways of *Scnn1b*-transgenic mice create a microaerophilic environment that promotes colonization of microaerophilic/anaerobic bacterial species, so microaerophilic culture conditions were used to harvest colony-forming units (CFUs) (26, 34, 35). To determine the effect of airway epithelial cell-specific HMGB1 deletion on spontaneous bacterial clearance, we harvested the BALF aseptically and counted the CFUs. The BALF collected from Cre^+^/WT and Cre^-^/WT mice were devoid of bacterial colonies (**Figure 6**). Although 3 of 14 Cre^-^/Tg+ mice had CFU counts (mean CFU ~340/ml), 15 of 21 Cre^+^/Tg+ mice had elevated CFU counts (mean CFU ~3461/ml), suggesting that airway epithelial cell-specific HMGB1 deficiency delays bacterial clearance in Tg+ mice (**Figure 6**).

### HMGB1 deletion in airway epithelial cells does not alter the airway epithelial cell composition

**Figure 7:**
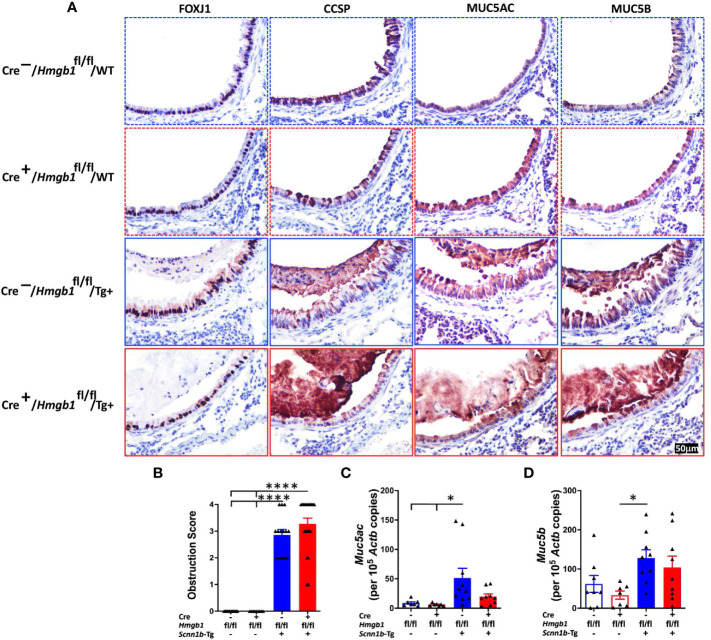
Airway epithelial cell-specific deletion of HMGB1 does not alter the airway epithelial cell composition and mucoobstruction. **(A)** Immunostaining of FOXJ1, CCSP, MUC5AC, and MUC5B in lung sections were performed to check the epithelial cell composition of the four groups (Cre^-^/WT [blue dotted border], Cre^+^/WT [red dotted border], Cre^-^/Tg+ [blue solid border], and Cre^+^/Tg+ [red solid border] mice). **(B)** Semiquantitative assessment for mucoobstruction from AB/PAS-stained left lung sections. Absolute quantification of *Muc5ac* mRNA **(C)** and *Muc5b* mRNA **(D)** in lung tissues. Different groups are shown as: Cre^-^/WT (blue open bar), Cre^+^/WT (red open bar), Cre^-^/Tg+ (blue solid bar), and Cre^+^/Tg+ (red solid bar). Error bars represent Mean ± SEM. One-way ANOVA followed by Tukey’s *post hoc* test was used for the statistical analysis. *p < 0.05, ****p < 0.0001.

Epithelial mesenchymal transition (EMT) is implicated in bronchial remodeling in asthma and chronic obstructive pulmonary disease (COPD), and HMGB1 can induce EMT in human airway epithelial cells (43, 44). To determine the effect of airway epithelial cell-specific HMGB1 deletion on airway epithelium in Tg+ lung disease, we compared the epithelial cell composition in the four experimental groups. The proportions of FOXJ1+ ciliated cells (**Figure 7A**), CCSP+ club cells (**Figure 7A**), MUC5AC+ mucous cells (**Figure 7A**), MUC5B+ mucous cells (**Figure 7A**), KRT5+ basal cells (**Supplemental Figure 3**), and KI-67+ proliferating airway epithelial cells (**Supplemental Figure 3**) were comparable between the Cre^-^/WT and Cre^+^/WT mice. As compared with Cre^-^/WT and Cre^+^/WT, the Cre^-^/Tg+ had an increased proportion of KI-67+ proliferating airway epithelial cells and MUC5B+ mucous cells. However, the Cre^-^/Tg+ and Cre^+^/Tg+ had a comparable composition of airway epithelial cells (**Figure 7A** and **Supplemental Figure 3**). These data suggest that the loss of HMGB1 in the airway epithelial cells does not alter the airway epithelial cell composition in WT as well as Tg+ mice.

To determine the effect of airway epithelial cell-specific HMGB1 deletion on mucoobstruction, semiquantitative assessment for luminal contents of AB/PAS-stained obstructed airways was performed (**Supplemental Figure 4**). As expected, while the Cre^-^/WT and Cre^+^/WT mice did not show any mucoobstruction in the airways, the Cre^-^/Tg+ exhibited a significant degree of mucoobstruction (**Figure 7B**). The degree of muco-obstructive score were comparable between Cre^-^/Tg+ and Cre^+^/Tg+ mice (**Figure 7B**). Consistent with the muco-obstructive scoring data, as compared to the Cre^-^/WT and Cre^+^/WT mice, the transcript levels for gel-forming mucins, i.e., *Muc5ac* and *Muc5b* were elevated in the Cre^-^/Tg+ mice (**Figures 7C, D**). The expression levels of *Muc5ac* and *Muc5b* were comparable between Cre^-^/Tg+ and Cre^+^/Tg+ mice (**Figures 7C, D**).

Next, we analyzed the expression levels of mucous cell metaplasia-relevant genes including *Il5*, *Il13*, *Il33*, *Clca1*, *Slc26a4*, *Agr2*, *Ear11*, and *Chi3l4* (**Supplemental Figure 5**). Consistent with the muco-obstructive scores, mucin immunostaining, and mucin gene expression, while the level of these transcripts were elevated in Cre^-^/Tg+ mice (**Supplemental Figure 5**), their expression levels were comparable between Cre^-^/Tg+ and Cre^+^/Tg+ mice (**Supplemental Figure 5**).

## Discussion

HMGB1 levels are elevated in the sputum of CF patients (18, 45). The *Scnn1b*-Tg+ (Tg+) mouse, a model of human CF-like lung disease, also exhibits elevated levels of HMGB1 in BALF (19). However, the source and the specific functions of the secreted HMGB1 in the lung airspaces remain unknown. Because epithelial cells are known to overexpress HMGB1 in airway lung diseases (23, 46, 47), we hypothesized that the airway epithelial cells are the primary producers of BALF HMGB1 and that HMGB1 modulates mucoinflammatory responses in the airways of Tg+ mice. Accordingly, we generated airway epithelial cell-specific HMGB1-deficient Tg+ mice and investigated the alterations in muco-inflammatory responses.

This study attempted to specifically address the following key questions: 1) Does airway epithelial cell-specific deletion of HMGB1 affect the normal development and constitution of airway epithelium in wild-type mice? 2) What is the source of HMGB1 in the BALF of Tg+ mice? 3) Does airway epithelial cell-specific deletion of HMGB1 affect the airway epithelial remodeling and lung inflammation in Tg+ mice? 4) Does airway epithelial cell-specific deletion of HMGB1 affect bacterial clearance in Tg+ mice? To answer these questions, we compared airway epithelial cell-specific HMGB1-deficient WT and Tg+ mice with their HMGB1-sufficient counterparts.

First, we assessed the effect of airway epithelial cell-specific HMGB1 deficiency on the composition of airway epithelial cells including goblet cells that are specialized for synthesis and secretion of mucins (48), ciliated cells that propel airway surface liquid through the coordinated ciliary beating (49), club cells that mainly reside in the bronchiolar epithelium and play several protective roles (50), and basal cells that present under the airway epithelial layer and function as progenitors of ciliated and secretory cells (51). Although the germ-line deletion of HMGB1 results in embryonic lethality (37, 38), airway epithelium-specific HMGB1 deficiency did not result in any developmental abnormalities. The composition of epithelial cells, i.e., ciliated cells, club cells, mucous cells, and basal cells, was comparable between HMGB1-deficient WT and HMGB1-sufficient WT mice. These data suggest that airway epithelial cell-specific deletion of HMGB1 does not affect the normal development and constitution of airway epithelium.

HMGB1 is released as an alarmin from either the airway epithelial cells (4, 5), alveolar epithelial cells (52), or other immune cells (53–55) in stressed lungs. While the BALF from HMGB1-sufficient WT mice contained HMGB1 protein, BALF from HMGB1-deficient WT mice had diminished levels of HMGB1 protein suggesting that the airway epithelial cells are the likely source of extracellular HMGB1 in the healthy lungs. However, the BALF HMGB1 levels were comparable between HMGB1-sufficient Tg+ and HMGB1-deficient Tg+ mice. These data indicate that the airway epithelial cells are one of the main sources of HMGB1 in the BALF of WT mice, but other cell types including, alveolar epithelial cells and recruited immune cells, are the likely producers of excessive amount of HMGB1 into the airspaces of Tg+ mice.

MUC5AC and MUC5B are two major airway gel-forming mucins that contribute to mucus obstruction in CF (56). Absence of MUC5B, not MUC5AC, in Tg+ mouse reduces airway mucus obstruction suggesting that the MUC5B, in particular, contributes to the muco-obstructive phenotype in these mice (57). Our previous studies also reported a strong association between MUC5AC and MUC5B expression and mucus obstruction (35, 58). HMGB1 has been reported to upregulate the expression of MUC5AC and MUC5B in human airway epithelial cells (59). Despite the airway epithelial cell-specific deletion of HMGB1, the BALF HMGB1 contents remained comparable in Cre^+^/Tg+ and Cre^-^/Tg+ mice. Therefore, it was not surprising to notice that the intensity of AB/PAS staining, MUC5AC/MUC5B immunostaining, and *Muc5ac/Muc5b* expression was also comparable between Cre^+^/Tg+ and Cre^-^/Tg+ mice. We are planning additional experiments in inducible systemic HMGB1-deficient Tg+ mice to determine whether the reduced HMGB1 can cause any reduction in the expression levels of gel-forming mucins and mucous cell metaplasia.

Mucous cell metaplasia is a consistent feature of Tg+ airways (25, 35). Inhibition of HMGB1 has been shown to reduce mucous cell metaplasia in mice with chronic allergic asthma, suggesting a role for HMGB1 in mucin production and mucous cell metaplasia (22). On the contrary, in the current study, the airway epithelial cell-specific HMGB1 deficiency did not reduce MUC5B-stained epithelial cells in Tg+ mice. Additionally, HMGB1 is known to promote proliferation in a variety of lung cells including airway epithelial cells (60). However, the extent of KI-67 staining of airway epithelial cells was comparable between HMGB1-sufficient and HMGB1-deficient Tg+ mice. These data suggest that the HMGB1 deficiency does not affect the mucous cell metaplasia and the airway epithelial cell proliferation in Tg+ mice.

HMGB1 has been shown to induce airway inflammation in muco-obstructive lung diseases (5). However, the specific mechanisms and functions of HMGB1 in regulating pulmonary airway inflammation remain unknown. HMGB1 has been reported to induce inflammatory cell recruitment (61, 62). For instance, intratracheal instillation of HMGB1 induced the lung neutrophilic inflammation through upregulating IL-1β, TNF-α, and MIP-2 (63). Counterintuitively, our data reveal that the airway epithelial cell-specific HMGB1 deficiency does not decrease inflammatory cell recruitment into the lung airspaces, instead it triggers the recruitment of macrophages, neutrophils, and eosinophils in the Tg+ mice. Along similar lines, airway epithelial cell-specific deletion of HMGB1 altered the levels of inflammatory mediators in the airspaces of Tg+ mice. For instance: 1) several immune cell-specific chemoattractants were significantly increased in the BALF of Cre^+^/Tg+ mice; and 2) pro-inflammatory cytokine, TNF-α, was present at a strikingly higher level in the BALF of Cre^+^/Tg+ mice. Together, these data suggest that the airway epithelial cell-specific deletion of HMGB1 promotes lung inflammation in Tg+ mice. However, further investigations are is needed to understand the molecular mechanisms involved.

The Tg+ mice are susceptible to spontaneous bacterial infection, a likely outcome of airway mucus obstruction, which is cleared significantly within four weeks of age (26, 31). Based on a previous report that demonstrated a beneficial effect of HMGB1 inhibition on bacterial clearance in CFTR^-/-^ mice (21), we anticipated that airway epithelial cell-specific HMGB1-deficient Tg+ mice will have a better bacterial clearance compared to HMGB1-sufficent Tg+ mice. Counterintuitively, we found that ablation of HMGB1 in airway epithelial cells significantly compromised bacterial clearance in Tg+ mice. This finding, however, is in line with previous reports that suggested a protective role of HMGB1 against bacterial infection (64, 65). Since HMGB1 deficiency in Tg+ mice did not compromise neutrophil recruitment and did not exaggerate mucus obstruction, we speculate that other unknown mechanisms are likely involved.

The current study leaves some knowledge gaps uncovered. First, while our exclusive focus in this study was on juveniles, the HMGB1 deletion may have different responses in neonates and adults. Second, while our data demonstrate that the airway epithelial cells are the primary producers of HMGB1 in the healthy airspaces, the additional cellular sources for the secreted HMGB1 into the stressed airspaces of Tg+ mice remain unknown. Third, the responsiveness of airway epithelial cell-specific HMGB1-deficient mice to additional challenges, i.e., air-borne pollutants or inhaled pathogens, remains unexplored. Finally, the mechanism underlying the increased protein leak and increased cellular recruitment into the airspaces of airway epithelial cell-specific HMGB1-deficient Tg+ mice remains elusive.

In conclusion, airway epithelial cell-specific deletion of HMGB1 significantly worsened the inflammatory manifestations in Tg+ mice including increased inflammatory cells recruitment, elevated levels of inflammatory mediators, and impaired bacterial clearance. The deficiency of HMGB1 in airway epithelial cells did not alter some hallmark features of Tg+ lung disease including mucus obstruction and airway epithelial remodeling. Taken together, these data indicate that airway epithelial cell-specific HMGB1 significantly modulates the inflammatory immune processes associated with muco-obstructive lung diseases.

## Data availability statement

The original contributions presented in the study are included in the article/**Supplementary Material**. Further inquiries can be directed to the corresponding author.

## Ethics statement

The animal study was reviewed and approved by LSU-IACUC.

## Author contributions

YM and YS conceived and designed the study. YM and YS performed the experiments. SP performed the histopathological analyses. YM, SP, and YS wrote and reviewed the manuscript for intellectual contents. All authors contributed to the article and approved the submitted version.

## Supplementary material

Supplementary Figure 1 Differential cell counts and percentage of BALF immune cells. The BALF immune cell counts and their corresponding percentages are shown. Different groups are shown as: Cre^-^/WT (blue open bar), Cre^+^/WT (red open bar), Cre^-^/Tg+ (blue solid bar), and Cre^+^/Tg+ (red solid bar). Error bars represent Mean ± SEM. One-way ANOVA followed by Tukey’s *post hoc* test was used for the statistical analysis. **p* < 0.05, ***p* < 0.01, ****p* < 0.001, *****p* < 0.0001.

Supplementary Figure 2 Expression analyses of apical junction complex-associated mRNAs. Absolute quantification of *Ocln* mRNA, *Tjp1* mRNA, *Ctnn1b* mRNA, and *Cdh1* mRNA in lung tissues from Cre^-^/WT (blue open bar), Cre^+^/WT (red open bar), Cre^-^/Tg+ (blue solid bar), and Cre^+^/Tg+ (red solid bar). Error bars represent Mean ± SEM. One-way ANOVA followed by Tukey’s *post hoc* test was used for the statistical analysis.

Supplementary Figure 3 Immunostaining of KRT5 and KI-67. Immunostaining of KRT5 and KI-67 confirms that airway epithelial cell-specific deletion of HMGB1 does not alter the airway epithelial cell composition. Red solid arrow indicates the positively stained cells in Cre^-^/WT [blue dotted border], Cre^+^/WT [red dotted border], Cre^-^/Tg+ [blue solid border], and Cre^+^/Tg+ [red solid border] mice.

Supplementary Figure 4 AB/PAS staining. AB/PAS staining indicate comparable staining of the epithelial cells and the luminal contents. Red solid arrow indicates the AB/PAS-stained cells in Cre^-^/Tg+ and Cre^+^/Tg+ mice.

Supplementary Figure 5 Expression analyses of mucous cell metaplasia-relevant mRNAs. Airway epithelial cell-specific deletion of HMGB1 does not alter the gene expression relevant to mucous cell metaplasia. Absolute quantification of *Il5* mRNA **(A)**, *Il13* mRNA **(B)**, *Il33* mRNA **(C)**, *Clca1* mRNA **(D)**, *Slc26a4* mRNA **(E)**, *Agr2* mRNA **(F)**, *Ear11* mRNA **(G)**, and *Chi3l4* mRNA **(H)** in lung tissues from Cre^-^/WT (blue open bar), Cre^+^/WT (red open bar), Cre^-^/Tg+ (blue solid bar), and Cre^+^/Tg+ (red solid bar). Error bars represent Mean ± SEM. One-way ANOVA followed by Tukey’s *post hoc* test was used for the statistical analysis. **p* < 0.05, ***p* < 0.01.
