# Supplementary figures and images for: Airway epithelial cell-specific deletion of HMGB1 exaggerates inflammatory responses in mice with muco-obstructive airway disease
Source: Front Immunol. 2023 Jan 19;13:944772. doi: 10.3389/fimmu.2022.944772 (PMC9892197; doi:10.3389/fimmu.2022.944772)

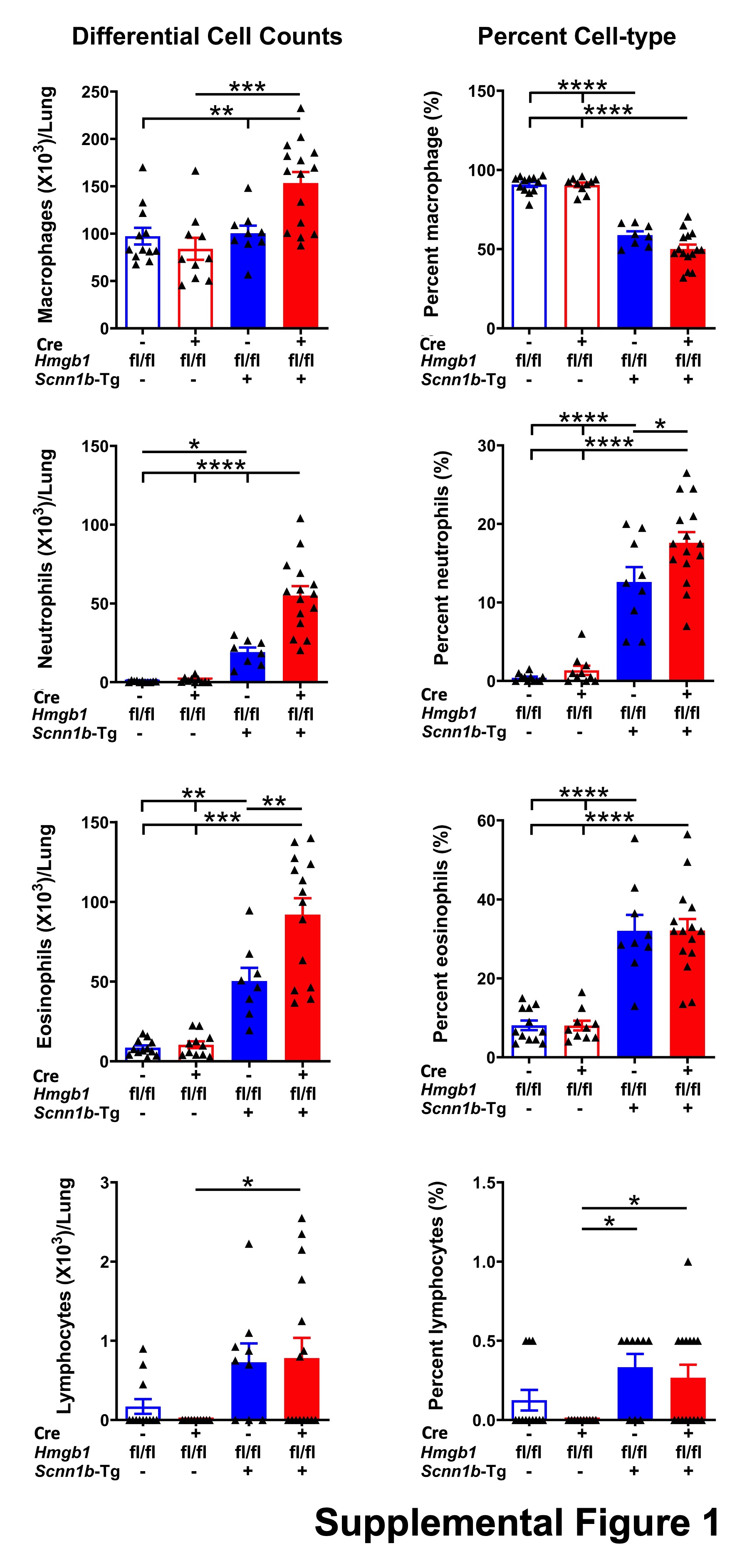

Supplement: Supplementary Figure 1 — Differential cell counts and percentage of BALF immune cells. The BALF immune cell counts and their corresponding percentages are shown. Different groups are shown as: Cre-/WT (blue open bar), Cre+/WT (red open bar), Cre-/Tg+ (blue solid bar), and Cre+/Tg+ (red solid bar). Error bars represent Mean ± SEM. One-way ANOVA followed by Tukey’s post hoc test was used for the statistical analysis. *p < 0.05, **p < 0.01, ***p < 0.001, ****p < 0.0001. [file Image_1.jpg]

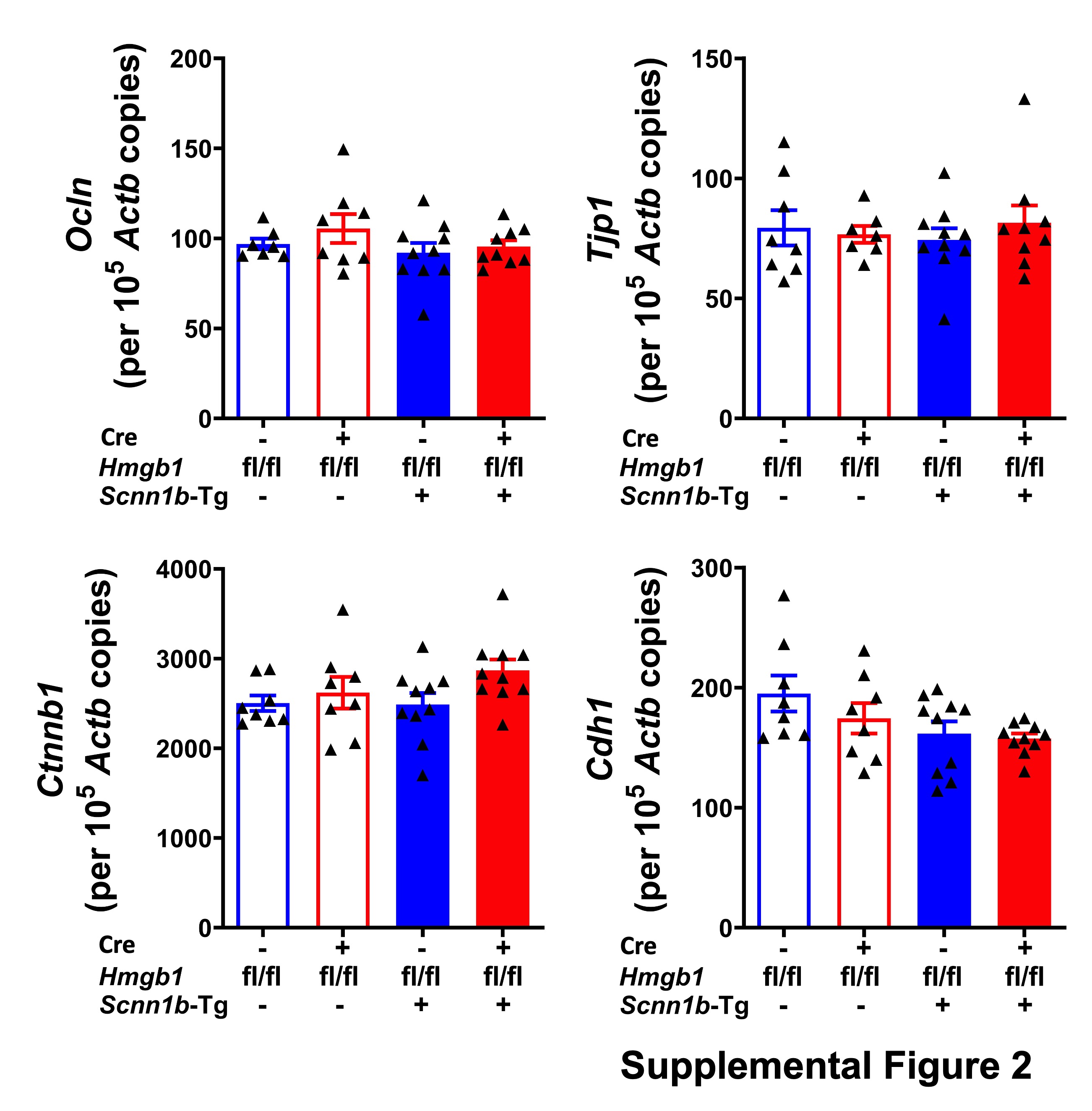

Supplement: Supplementary Figure 2 — Expression analyses of apical junction complex-associated mRNAs. Absolute quantification of Ocln mRNA, Tjp1 mRNA, Ctnn1b mRNA, and Cdh1 mRNA in lung tissues from Cre-/WT (blue open bar), Cre+/WT (red open bar), Cre-/Tg+ (blue solid bar), and Cre+/Tg+ (red solid bar). Error bars represent Mean ± SEM. One-way ANOVA followed by Tukey’s post hoc test was used for the statistical analysis. [file Image_2.jpg]

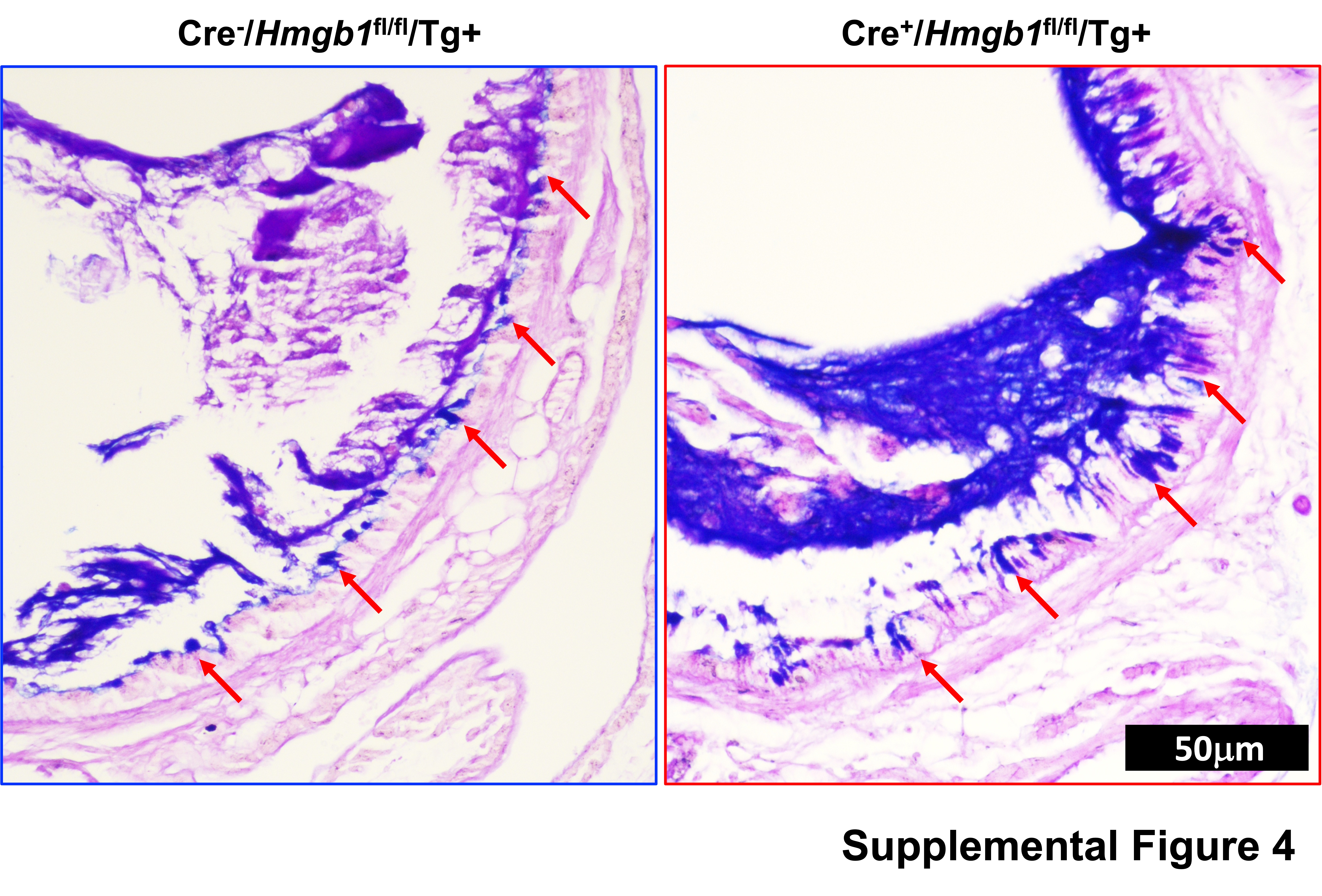

Supplement: Supplementary Figure 4 — AB/PAS staining. AB/PAS staining indicate comparable staining of the epithelial cells and the luminal contents. Red solid arrow indicates the AB/PAS-stained cells in Cre-/Tg+ and Cre+/Tg+ mice. [file Image_4.jpg]

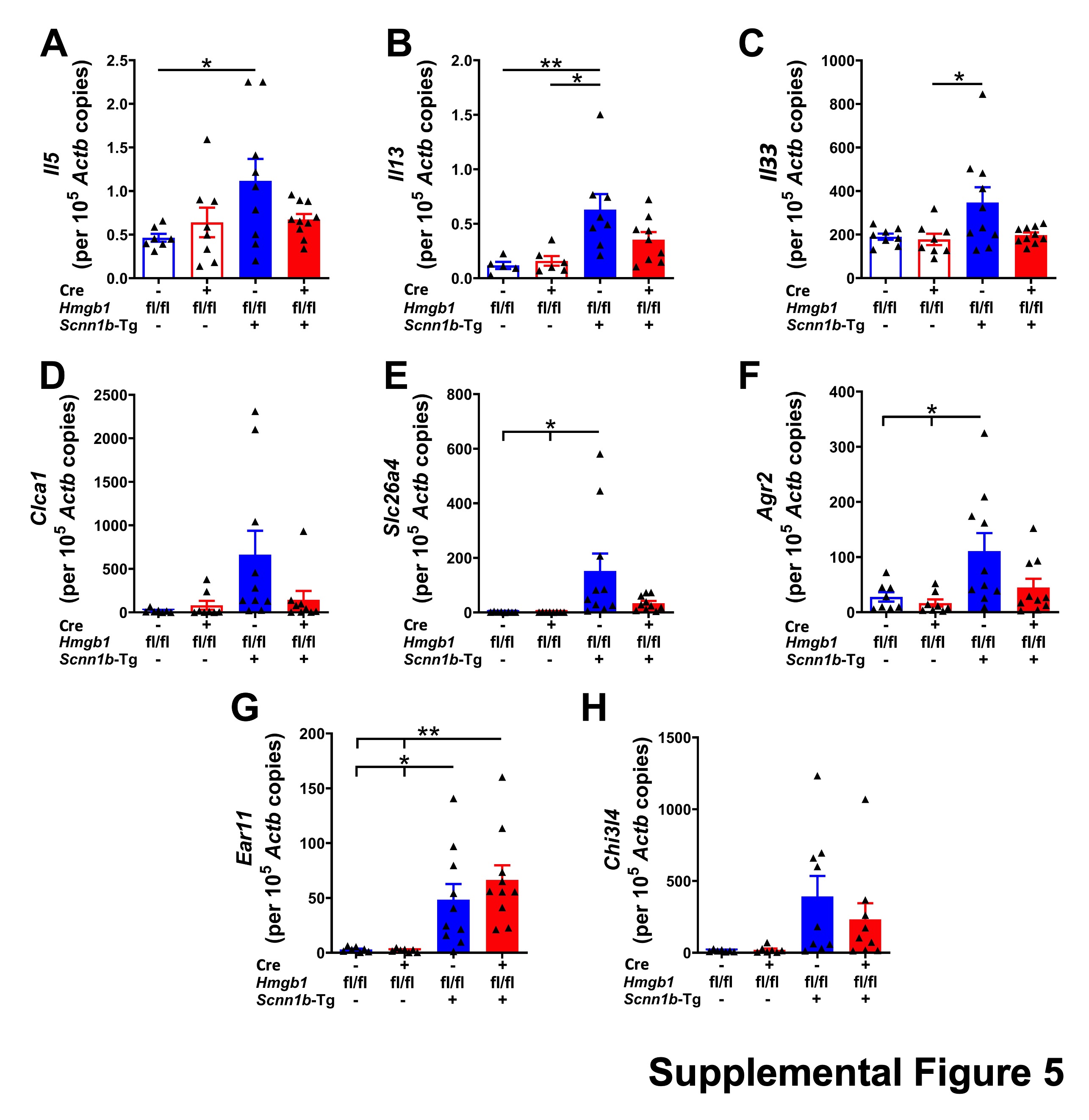

Supplement: Supplementary Figure 5 — Expression analyses of mucous cell metaplasia-relevant mRNAs. Airway epithelial cell-specific deletion of HMGB1 does not alter the gene expression relevant to mucous cell metaplasia. Absolute quantification of Il5 mRNA (A), Il13 mRNA (B), Il33 mRNA (C), Clca1 mRNA (D), Slc26a4 mRNA (E), Agr2 mRNA (F), Ear11 mRNA (G), and Chi3l4 mRNA (H) in lung tissues from Cre-/WT (blue open bar), Cre+/WT (red open bar), Cre-/Tg+ (blue solid bar), and Cre+/Tg+ (red solid bar). Error bars represent Mean ± SEM. One-way ANOVA followed by Tukey’s post hoc test was used for the statistical analysis. *p < 0.05, **p < 0.01. [file Image_5.jpg]
