# Supplementary material for: Airway epithelial cell-specific deletion of HMGB1 exaggerates inflammatory responses in mice with muco-obstructive airway disease
Source: Front Immunol. 2023 Jan 19;13:944772. doi: 10.3389/fimmu.2022.944772 (PMC9892197; doi:10.3389/fimmu.2022.944772)
Supplement: Supplementary file 6 [file Table_1.docx]

**Supplemental Table 1:** Primer sequences for PCR genotyping and RT-PCR.

| **No** | **Gene Symbol** | **Forward Sequence** | **Reverse Sequence** |
| --- | --- | --- | --- |
| **PCR Genotyping Primers** | | | |
| 1 | *Scnn1b (*genomic DNA*; gDNA)* | CTTCCAAGAGTTCAACTACCG | TCTACCAGCTCAGCCACAGTG |
| 2 | *Hmgb1 (gDNA)* | TGTCATGCCACCCTGAGCAGTT | TGTGCTCCTCCCGGCAAGTT |
| 3 | *CC10-Cre (gDNA)* | TCTGATGAAGTCAGGAAGAACC | GAGATGTCCTTCACTCTGATTC |
| 4 | *CC10-Cre Internal Control (gDNA)* | TGCCAGAGATTGTTCTAGAAAACAA | GGCACAATGATGTTAATGACGTAAA |
| **RT-PCR Primers** | | | |
| 1 | *Actb (mRNA)* | GGCTGTATTCCCCTCCATCG | GGGGTACTTCAGGGTCAGGA |
| 2 | *Muc5ac (mRNA)* | CCATGCAGAGTCCTCAGAACAA | TTACTGGAAAGGCCCAAGCA |
| 3 | *Muc5b (mRNA)* | CTGCACAGATACGGAGGACA | TGGACACAGGCATCCAAGTA |
| 4 | *Il5 (mRNA)* | GCCAAAAAGAGAAGTGTGGCG | CTCAGCCTCAGCCTTCCATT |
| 5 | *Il13 (mRNA)* | ATCACACAAGACCAGACTCCC | CTCTGGGTCCTGTAGATGGC |
| 6 | *Il33 (mRNA)* | GCGTCATTCCTCTGGAAACC | TGGTCACACGTGGTTTTGAA |
| 7 | *Clca1 (mRNA)* | AAGCAGTGAGGTGTTCAGCA | CAGTCCCGTTACTCTGTCGAT |
| 8 | *Slc26a4 (mRNA)* | GACTGTAAAGACCCTCTTGATCTGA | GGAAGCAAGTCTACGCATGG |
| 9 | *Agr2 (mRNA)* | AGCCCAGATTTGCCATGGAG | CTGAGGTAGTTTGGGCCGAG |
| 10 | *Ear11 (mRNA)* | ACTGGGAAACATGGGTCTGG | TGCTGGATGTCAAACCACCG |
| 11 | *Chi3l4 (mRNA)* | CCACTTTGAACCACATTCCAAGG | GAGAGACTGAGACAGTTCAGGG |
| 12 | *Ocln (mRNA)* | AAGTCAACACCTCTGGTGCC | TACCATTGCTGCTGTACCGA |
| 13 | *Tjp1 (mRNA)* | GACGGTGGCGTGAGGAG | CATTGCTGTGCTCTTAGCGG |
| 14 | *Ctnnb1 (mRNA)* | GAATGAAGGCGTGGCAACAT | TGGTCAGCTCGACTGAAAGC |
| 15 | *Cdh1 (mRNA)* | AACCCAAGCACGTATCAGGG | GAGTGTTGGGGGCATCATCA |
