# Supplementary material for: Airway epithelial cell-specific deletion of HMGB1 exaggerates inflammatory responses in mice with muco-obstructive airway disease
Source: Front Immunol. 2023 Jan 19;13:944772. doi: 10.3389/fimmu.2022.944772 (PMC9892197; doi:10.3389/fimmu.2022.944772)
Supplement: Supplementary file 7 [file Table_2.docx]

**Supplemental Table 2**: Cytokine in BALF (picograms per milliliter)

| **Cytokine** | **Cre^-^/WT** | **Cre^+^/WT** | **Cre^-^/Tg+** | **Cre^+^/Tg+** | **LOD** |
| --- | --- | --- | --- | --- | --- |
| IL-4 | 0.34 ± 0.22 | 0.44 ± 0.15 | 9.90 ± 2.94 | **61.57 ± 13.81** | 0.02 |
| IL-5 | 1.41 ± 0.00 | 1.41 ± 0.00 | 35.07 ± 10.95 | **69.67 ± 11.16** | 1.42 |
| RANTES | 2.00 ± 0.18 | 2.49 ± 0.24 | 2.04 ± 0.12 | 2.40 ± 0.12 | 1.39 |
| MIP-1α | 24.55 ± 8.50 | 27.21 ± 8.88 | 51.45 ± 13.86 | **187.37 ± 12.23** | 8.67 |
| MIP-1β | 6.99 ± 1.65 | 0.25 ± 0.00 | 12.43 ± 1.98 | **53.48 ± 6.24** | 0.26 |
| KC | 2.89 ± 0.90 | 3.35 ± 0.52 | 78.96 ± 15.39 | **366.57 ± 92.11** | 0.42 |
| IL-6 | 0.46 ± 0.00 | 0.50 ± 0.04 | 5.55 ± 5.09 | 5.17 ± 1.75 | 0.47 |
| TNF-α | 0.20 ± 0.06 | 0.20 ± 0.06 | 0.32 ± 0.12 | **4.39 ± 1.10** | 0.15 |
| MIP-2 | 37.92 ± 7.15 | 23.50 ± 0.00 | 88.39 ± 24.39 | **684.08 ± 101.33** | 23.51 |
| G-CSF | 2.10 ± 0.80 | 2.58 ± 1.28 | 86.23 ± 14.31 | **313.11 ± 43.87** | 0.02 |
| IL-7 | 2.02 ± 0.58 | 1.03 ± 0.50 | 1.41 ± 0.53 | 0.27 ± 0.16 | 0.01 |
| IL-10 | 5.79 ± 2.07 | 13.39 ± 4.12^π #^ | 0.37 ± 0.00^π^ | 2.37 ± 0.88^#^ | 0.38 |
| MCP-1 | 11.27 ± 2.45 | 6.83 ± 0.00 | 21.94 ± 4.52 | **45.55 ± 6.26** | 6.84 |
| IL-2 | 3.69 ± 0.24* | 5.77 ± 0.68*^π^^Ф^ | 3.52 ± 0.29^π^ | 3.49 ± 0.16^Ф^ | 2.29 |
| IL-9 | 98.12 ± 21.93 | 74.64 ± 27.82 | 71.29 ± 23.80 | 80.76 ± 19.92 | 16.46 |
| IL-15 | 1.76 ± 1.00* | 3.18 ± 1.03 | 2.26 ± 0.94^Ѱ^ | 6.54 ± 1.16*^Ѱ^ | 0.19 |
| IL-1α | 11.36 ± 3.47* | 36.47 ± 8.52*^π^ | 10.72 ± 4.84^π^ | 23.76 ± 3.61 | 0.08 |
| IL-1β | 0.72 ± 0.34 | 0.79 ± 0.35 | 0.25 ± 0.00 | 1.68 ± 0.56 | 0.25 |
| IFN-λ | 1.71 ± 0.62 | 6.31 ± 2.31 | 1.71 ± 0.62 | 4.74 ± 1.49 | 1.10 |
| IP-10 | 5.78 ± 1.44 | 8.63 ± 1.41 | 12.73 ± 2.71 | **22.35 ± 1.65** | 2.07 |

Two values sharing identical designations (* or ^π^ or ^#^ or ^Ѱ^ or ^Ф^) within a row indicate significant differences. Values in bold indicate that the Cre^+^/Tg+ value is significantly different than the other three groups, i.e., Cre^-^/WT, Cre^+^/WT, and Cre^-^/Tg+. Values that were below the LOD [< out-of-range (OOR)] were assigned value 0.01 unit less than the LOD. n = 7-9 in each group, *p* < 0.05. LOD means lower limit of detection or lowest value obtained in the assay.
